# Supplementary material for: A Likelihood Approach for Real-Time Calibration of Stochastic Compartmental Epidemic Models
Source: PLoS Comput Biol. 2017 Jan 17;13(1):e1005257. doi: 10.1371/journal.pcbi.1005257 (PMC5240920; doi:10.1371/journal.pcbi.1005257)
Supplement: S1 File — (TAR.GZ) [file pcbi.1005257.s014.tar.gz › HSPH_Online-SI-Revision/MSS10/n10-extreme/n10-extreme_NEW_table-4.pdf]

| | | | | | | | | | | | | | | | | | | | | | | | | | | | |
|---|---|---|---|---|---|---|---|---|---|---|---|---|---|---|---|---|---|---|---|---|---|---|---|---|---|---|---|
| 1 | R0 | {2.68692, 12.5884, 1.} | {1.92433, 7.25444, 0.14} | {{1.47608, 4.02511, 0.}, {2.52588, 12.2207, 1.}} | {28.5071, 43.1124, 88.} | inf | 652. | 383.349 | {214.489, 638.8} | 41.5762 | 0.270025 | newly | 547. | 531.02 | {444., 632.} | 11.9598 | 0.0536478 | Reff | 2.38787 | 1.75609 | {1.38447, 2.24869} | 26.5786 | ar | 9089. | 7439.57 | {5970., 8955.} | 18.1783 |
| 2 | R0 | {1.79802, 12.5412, 1.} | {1.76101, 10.7409, 2.04} | {{1.24751, 3.07157, 0.}, {2.01498, 14.4607, 3.}} | {17.5034, 33.9462, 162.} | inf | 658. | 596.805 | {149.508, 850.752} | 40.007 | 0.251754 | newly | 408. | 439.87 | {335., 506.} | 15.6544 | 0.0668034 | Reff | 1.51609 | 1.48551 | {1.10555, 1.67426} | 14.777 | ar | 7412. | 6636.87 | {3621., 8101.} | 20.8973 |
| 3 | R0 | {2.6174, 10.1105, 0.1} | {2.82351, 9.72235, 0.09} | {{2.17292, 6.16453, 0.}, {2.99092, 10.5991, 0.}} | {12.6142, 7.77416, 174.} | inf | 487. | 484.78 | {325.028, 580.448} | 14.2916 | 0.0691097 | newly | 540. | 626.48 | {520., 727.} | 18.237 | 0.0712849 | Reff | 2.41377 | 2.60236 | {2.04136, 2.74015} | 12.122 | ar | 9109. | 9212.05 | {8427., 9419.} | 3.47689 |
| 4 | R0 | {2.27532, 14.3149, 0.1} | {2.01194, 11.9348, 1.7} | {{1.87584, 10.2502, 1.}, {2.0968, 13.1443, 2.}} | {11.5757, 16.6269, 1604.} | inf | 1121. | 962.067 | {793.952, 1071.71} | 14.3416 | 0.0749464 | newly | 626. | 656.04 | {599., 723.} | 7.20447 | 0.0300535 | Reff | 1.73038 | 1.56023 | {1.46705, 1.61464} | 9.83295 | ar | 8478. | 7916.96 | {7450., 8235.} | 6.6176 |
| 5 | R0 | {2.96649, 18.0674, 2.} | {2.61558, 14.7453, 1.79} | {{1.93419, 8.56054, 0.}, {2.96398, 17.7348, 2.}} | {11.829, 18.3871, 10.5} | inf | 1477. | 1209.08 | {755.022, 1524.77} | 19.4579 | 0.11761 | newly | 689. | 716.84 | {671., 765.} | 6.13062 | 0.0261353 | Reff | 2.25186 | 2.04194 | {1.62051, 2.25568} | 9.44894 | ar | 9476. | 8843.57 | {7816., 9402.} | 6.67402 |
| 6 | R0 | {2.94778, 16.0002, 0.1} | {1.58805, 4.96205, 0.21} | {{1.44762, 3.83841, 0.}, {1.93419, 8.56054, 0.}} | {46.1273, 68.9876, 292.} | inf | 820. | 295.259 | {165.252, 416.212} | 64.7354 | 0.491826 | newly | 489. | 566.65 | {399., 738.} | 24.8773 | 0.0974243 | Reff | 2.5348 | 1.4445 | {1.33086, 1.71873} | 43.0132 | ar | 9321. | 6070.02 | {5367., 7577.} | 34.878 |
| 7 | R0 | {2.17562, 11.7184, 1.} | {2.55503, 12.2988, 1.6} | {{1.24751, 2.5364, 0.}, {2.99911, 15.6207, 2.}} | {35.2144, 39.286, 102.} | inf | 569. | 661.285 | {108.436, 897.122} | 49.262 | 0.276218 | newly | 469. | 533.92 | {405., 599.} | 24.8657 | 0.104478 | Reff | 1.95066 | 2.24826 | {1.17598, 2.61275} | 31.7888 | ar | 8345. | 8187.85 | {3776., 9419.} | 20.315 |
| 8 | R0 | {2.7904, 17.8966, 2.} | {2.38633, 13.5001, 0.9} | {{1.44762, 4.00571, 0.}, {2.60786, 15.5928, 1.}} | {15.1185, 24.6947, 56.} | inf | 1183. | 867.287 | {279.368, 1061.83} | 26.7423 | 0.172266 | newly | 589. | 577.16 | {522., 627.} | 6.56706 | 0.0288233 | Reff | 2.25715 | 1.99436 | {1.31064, 2.17635} | 12.4376 | ar | 9285. | 8416.65 | {5636., 9128.} | 9.42714 |
| 9 | R0 | {2.51574, 16.376, 1.} | {2.78478, 18.2648, 1.97} | {{2.76849, 18.1089, 2.}, {2.86656, 18.9813, 2.}} | {12.3712, 14.7693, 99.} | inf | 1086. | 1325.67 | {1137.83, 1467.15} | 24.2867 | 0.099504 | newly | 556. | 619.53 | {562., 680.} | 12.7392 | 0.0520871 | Reff | 2.01712 | 2.16289 | {2.14901, 2.21297} | 8.74162 | ar | 8937. | 9186.09 | {9122., 9341.} | 4.17489 |
| 10 | R0 | {2.56405, 18.1787, 2.} | {1.48715, 5.90363, 0.79} | {{1.22544, 3.07157, 0.}, {2.33995, 14.464, 3.}} | {41.9998, 67.5244, 83.5} | inf | 985. | 333.461 | {105.851, 850.578} | 66.4456 | 0.601867 | newly | 476. | 449.41 | {253., 661.} | 23.1618 | 0.114933 | Reff | 2.09996 | 1.30338 | {1.1038, 1.94787} | 37.9332 | ar | 9005. | 4929.28 | {3214., 8560.} | 45.2606 |
| 11 | R0 | {2.475, 17.1709, 1.} | {1.26057, 3.49019, 0.05} | {{1.22544, 3.07157, 0.}, {1.28596, 3.98513, 0.}} | {49.0678, 79.6738, 97.} | inf | 1114. | 187.94 | {109.207, 253.701} | 83.1293 | 0.812945 | newly | 539. | 410.4 | {285., 551.} | 26.7829 | 0.155367 | Reff | 1.97134 | 1.12001 | {1.08575, 1.14069} | 43.1853 | ar | 8870. | 3778.26 | {3288., 4189.} | 57.4041 |
| 12 | R0 | {2.14974, 11.8619, 0.1} | {1.37115, 4.62845, 0.23} | {{1.21159, 2.5364, 0.}, {2.08003, 12.1899, 1.}} | {38.4562, 67.1419, 298.} | inf | 514. | 166.097 | {45.4778, 444.121} | 73.7496 | 0.686355 | newly | 376. | 272.18 | {131., 386.} | 30.9149 | 0.204469 | Reff | 1.93176 | 1.27279 | {1.14278, 1.88365} | 35.9251 | ar | 8414. | 4057.21 | {1904., 8241.} | 52.5521 |
| 13 | R0 | {2.20385, 14.4767, 0.1} | {1.29835, 3.8828, 0.04} | {{1.22544, 3.07157, 0.}, {1.28596, 3.98513, 0.}} | {42.0341, 75.6251, 132.} | inf | 1178. | 264.392 | {120.836, 311.336} | 79.6654 | 0.744096 | newly | 681. | 480.18 | {290., 665.} | 30.047 | 0.182515 | Reff | 1.66898 | 1.09589 | {1.04167, 1.09015} | 34.9175 | ar | 8347. | 3935. | {3137., 4329.} | 53.3976 |
| 14 | R0 | {2.35671, 13.8357, 1.} | {2.13798, 10.256, 0.93} | {{1.25474, 3.26445, 0.}, {2.96398, 17.7348, 2.}} | {32.9228, 51.7204, 99.} | inf | 1020. | 834.384 | {196.795, 1489.24} | 56.6001 | 0.361737 | newly | 683. | 692.7 | {533., 791.} | 12.0469 | 0.0537604 | Reff | 1.89291 | 1.7131 | {1.11435, 2.26445} | 27.0249 | ar | 8719. | 7146.35 | {4083., 9395.} | 24.8486 |
| 15 | R0 | {1.91368, 10.1082, 1.} | {2.33072, 16.5198, 1.96} | {{2.35866, 16.9062, 2.}, {2.35866, 16.9062, 2.}} | {22.4582, 64.5619, 96.} | inf | 746. | 1208.01 | {1164.41, 1286.31} | 63.025 | 0.211178 | newly | 600. | 597.44 | {555., 635.} | 3.9 | 0.0170488 | Reff | 1.56616 | 1.7983 | {1.79988, 1.82804} | 15.3979 | ar | 7486. | 8663.72 | {8623., 8813.} | 16.0796 |
| 16 | R0 | {2.48727, 14.882, 0.1} | {2.71503, 14.9887, 0.86} | {{2.36114, 11.6557, 0.}, {2.93371, 17.1903, 1.}} | {10.4872, 14.4833, 788.} | inf | 818. | 798.908 | {664.851, 905.375} | 9.51444 | 0.0432322 | newly | 517. | 509.63 | {456., 582.} | 8.21857 | 0.0358156 | Reff | 2.15174 | 2.35214 | {2.09363, 2.53114} | 10.1994 | ar | 8894. | 9143.7 | {8838., 9389.} | 3.20441 |
| 17 | R0 | {2.46795, 13.1915, 0.1} | {2.66447, 12.9171, 1.63} | {{1.74092, 5.58477, 0.}, {2.99911, 15.6207, 2.}} | {18.1932, 21.0941, 1564.} | inf | 685. | 722.051 | {318.161, 895.928} | 23.1397 | 0.111094 | newly | 499. | 561.94 | {498., 647.} | 13.7395 | 0.0542045 | Reff | 2.17698 | 2.33227 | {1.59953, 2.60272} | 16.4195 | ar | 8796. | 8883.77 | {7107., 9408.} | 7.97306 |
| 18 | R0 | {2.96603, 16.7543, 0.1} | {2.47671, 13.8573, 1.43} | {{1.93419, 8.56054, 0.}, {2.99088, 18.9406, 2.}} | {16.9715, 24.9923, 1372.} | inf | 1177. | 907.547 | {567.592, 1194.6} | 24.8379 | 0.13841 | newly | 657. | 596.41 | {534., 672.} | 10.6256 | 0.0496634 | Reff | 2.40011 | 2.06094 | {1.67622, 2.42932} | 14.6925 | ar | 9394. | 8730.46 | {7770., 9411.} | 7.17969 |
| 19 | R0 | {2.25216, 9.90465, 0.1} | {2.18611, 8.07505, 0.25} | {{1.737, 5.43578, 0.}, {2.6266, 11.0527, 1.}} | {9.86172, 24.4613, 302.} | inf | 358. | 289.613 | {171.103, 402.426} | 26.0369 | 0.146556 | newly | 390. | 396.13 | {301., 481.} | 15.2487 | 0.0678357 | Reff | 2.11343 | 2.06391 | {1.66057, 2.44513} | 9.44244 | ar | 8631. | 8258.36 | {7110., 9095.} | 6.10474 |
| 20 | R0 | {2.70837, 18.1923, 0.1} | {2.62502, 15.6099, 0.9} | {{2.27199, 11.6669, 0.}, {2.90165, 18.0404, 1.}} | {7.50183, 14.195, 820.} | inf | 1099. | 996.211 | {782.146, 1185.21} | 13.0021 | 0.0681931 | newly | 555. | 569.97 | {518., 612.} | 6.44865 | 0.028808 | Reff | 2.21924 | 2.17399 | {1.92028, 2.36659} | 5.95217 | ar | 9183. | 9019.36 | {8635., 9355.} | 2.88686 |
| 21 | R0 | {2.30288, 15.5877, 1.} | {2.11747, 13.0122, 0.} | {{2.11292, 12.9719, 0.}, {2.11292, 12.9719, 0.}} | {8.28128, 16.7042, 100.} | inf | 1079. | 893.083 | {843.576, 937.35} | 17.8486 | 0.0853637 | newly | 546. | 571.34 | {539., 602.} | 5.60073 | 0.0234339 | Reff | 1.82273 | 1.71511 | {1.70231, 1.72212} | 6.04049 | ar | 8674. | 8243.25 | {8137., 8329.} | 5.0543 |
| 22 | R0 | {2.82208, 10.7544, 0.1} | {2.38411, 9.27806, 0.43} | {{1.74592, 5.58477, 0.}, {2.99939, 13.7076, 1.}} | {18.2296, 28.1556, 448.} | inf | 295. | 259.456 | {135.368, 367.95} | 27.1536 | 0.143794 | newly | 339. | 323.33 | {246., 396.} | 14.2802 | 0.0661456 | Reff | 2.6838 | 2.27204 | {1.68945, 2.83237} | 17.7174 | ar | 9333. | 8488.41 | {7130., 9417.} | 9.42516 |
| 23 | R0 | {1.69923, 10.1366, 1.} | {1.24643, 3.12005, 0.} | {{1.22544, 2.5364, 0.}, {1.25474, 3.26445, 0.}} | {26.6471, 69.2199, 100.} | inf | 645. | 175.526 | {82.4327, 256.046} | 72.7866 | 0.600599 | newly | 502. | 419.98 | {226., 606.} | 26.7729 | 0.148152 | Reff | 1.4056 | 1.08955 | {1.06714, 1.1087} | 22.4852 | ar | 7055. | 3606.43 | {3200., 4066.} | 48.8812 |
| 24 | R0 | {2.64944, 19.054, 1.} | {2.73543, 19.2578, 1.01} | {{2.76795, 19.6041, 1.}, {2.76795, 19.6041, 1.}} | {5.34258, 4.47402, 1.} | inf | 1801. | 1841.83 | {1788.82, 1932.82} | 5.53184 | 0.0254395 | newly | 721. | 759.91 | {718., 803.} | 5.77947 | 0.0241737 | Reff | 1.75684 | 1.80026 | {1.79561, 1.83039} | 3.94263 | ar | 9147. | 9148.31 | {9126., 9258.} | 1.20356 |
| 25 | R0 | {2.53191, 10.9214, 0.1} | {1.81634, 5.52598, 0.06} | {{1.53501, 4.02511, 0.}, {1.9862, 6.28417, 0.}} | {28.262, 49.4023, 148.} | inf | 473. | 274.009 | {172.326, 364.692} | 42.0699 | 0.253789 | newly | 441. | 535.16 | {388., 671.} | 27.6916 | 0.105412 | Reff | 2.32379 | 1.70222 | {1.43742, 1.86078} | 26.748 | ar | 9009. | 7240.35 | {5908., 7997.} | 19.632 |
| 26 | R0 | {1.89155, 6.76944, 0.1} | {1.99482, 7.93249, 0.93} | {{1.24751, 2.5364, 0.}, {2.99911, 15.6207, 2.}} | {35.4236, 74.5306, 942.} | inf | 395. | 447.262 | {116.43, 851.758} | 72.4291 | 0.331027 | newly | 577. | 538.54 | {340., 720.} | 18.7106 | 0.0935851 | Reff | 1.71999 | 1.78207 | {1.16643, 2.56797} | 31.7169 | ar | 7348. | 6786.09 | {3578., 9378.} | 29.5235 |
| 27 | R0 | {2.63149, 15.6979, 0.1} | {2.52811, 12.6908, 0.08} | {{2.32891, 10.9956, 0.}, {2.96947, 15.963, 0.}} | {9.21405, 20.4905, 164.} | inf | 797. | 720.642 | {609.247, 890.13} | 15.1643 | 0.0722181 | newly | 482. | 548.33 | {490., 600.} | 13.9357 | 0.0556294 | Reff | 2.28124 | 2.20822 | {2.04831, 2.54658} | 7.91222 | ar | 9104. | 8899.43 | {8571., 9367.} | 3.45771 |
| 28 | R0 | {2.85141, 12.6992, 1.} | {2.28803, 8.11964, 0.37} | {{1.98388, 6.25659, 0.}, {2.79531, 11.6897, 1.}} | {19.7579, 36.0618, 73.} | inf | 622. | 399.335 | {239.201, 563.293} | 35.8117 | 0.213736 | newly | 530. | 570.24 | {448., 674.} | 16.7623 | 0.0677564 | Reff | 2.57824 | 2.11599 | {1.83731, 2.54815} | 17.935 | ar | 9317. | 8443.63 | {7794., 9232.} | 9.37673 |
| 29 | R0 | {2.54244, 12.9085, 0.1} | {1.772, 6.09048, 0.18} | {{1.24751, 2.5364, 0.}, {2.13505, 8.59564, 1.}} | {30.3033, 52.8181, 248.} | inf | 820. | 409.173 | {117.51, 616.28} | 50.1008 | 0.38083 | newly | 612. | 623.74 | {363., 767.} | 19.1242 | 0.0884398 | Reff | 2.19184 | 1.59214 | {1.15116, 1.90217} | 27.3603 | ar | 9034. | 6520.97 | {3542., 8368.} | 27.8175 |
| 30 | R0 | {2.10982, 11.7636, 1.} | {2.14178, 12.056, 0.73} | {{1.24751, 2.5364, 0.}, {2.64334, 17.2462, 1.}} | {21.5499, 41.3338, 27.} | inf | 762. | 752.338 | {144.16, 1097.73} | 38.279 | 0.240721 | newly | 521. | 530.53 | {403., 630.} | 13.0038 | 0.0626577 | Reff | 1.77373 | 1.78335 | {1.12223, 2.14969} | 18.4438 | ar | 8224. | 7535.47 | {3511., 9103.} | 17.9911 |
| 31 | R0 | {2.76312, 15.5396, 0.1} | {1.59692, 4.88721, 0.18} | {{1.44762, 3.83841, 0.}, {1.737, 5.43578, 0.}} | {42.7029, 68.5793, 262.} | inf | 1053. | 379.384 | {233.138, 447.452} | 65.1787 | 0.49388 | newly | 607. | 722.59 | {551., 888.} | 23.3756 | 0.0896135 | Reff | 2.27211 | 1.41257 | {1.30211, 1.53675} | 38.1669 | ar | 9220. | 6056.51 | {5500., 7106.} | 34.4264 |
| 32 | R0 | {2.21727, 15.5439, 0.1} | {2.37462, 19.3966, 0.} | {{2.37462, 19.3966, 0.}, {2.37462, 19.3966, 0.}} | {7.09672, 24.7857, 100.} | inf | 1094. | 1280.56 | {1234.26, 1322.94} | 17.0526 | 0.06823 | newly | 577. | 528.48 | {499., 561.} | 8.48527 | 0.0388984 | Reff | 1.72703 | 1.80529 | {1.79403, 1.81609} | 4.53163 | ar | 8359. | 8762.96 | {8686., 8839.} | 4.83264 |
| 33 | R0 | {2.72912, 10.8159, 0.1} | {2.67691, 12.334, 0.09} | {{2.22376, 9.35432, 0.}, {2.86001, 13.5542, 0.}} | {6.45824, 19.8534, 174.} | inf | 759. | 704.37 | {518.452, 819.879} | 11.0797 | 0.0550269 | newly | 733. | 579.15 | {523., 634.} | 20.9891 | 0.103408 | Reff | 2.39835 | 2.3646 | {2.00643, 2.52167} | 5.78507 | ar | 9196. | 9081.97 | {8529., 9320.} | 2.31394 |
| 34 | R0 | {2.20928, 10.1308, 1.} | {1.42731, 3.21027, 0.2} | {{1.24751, 1.89883, 0.}, {1.53501, 4.02511, 1.}} | {37.6168, 70.4126, 98.} | inf | 601. | 216.399 | {79.4917, 310.829} | 67.5429 | 0.540964 | newly | 568. | 656.68 | {325., 990.} | 40.4577 | 0.163765 | Reff | 1.96317 | 1.31897 | {1.15738, 1.42482} | 34.7293 | ar | 8388. | 4974.83 | {3597., 6109.} | 41.5733 |
| 35 | R0 | {2.10103, 15.2673, 2.} | {2.59795, 17.327, 1.86} | {{1.86868, 9.75343, 2.}, {2.86095, 19.9929, 2.}} | {30.722, 30.4618, 7.} | inf | 1074. | 1378.81 | {772.46, 1654.23} | 43.3836 | 0.19059 | newly | 563. | 647.55 | {600., 689.} | 17.6643 | 0.0723901 | Reff | 1.64364 | 1.93362 | {1.51763, 2.09711} | 23.3728 | ar | 8264. | 8691.78 | {7655., 9341.} | 14.0099 |
| 36 | R0 | {2.74914, 19.2775, 1.} | {1.53014, 5.24032, 0.36} | {{1.44762, 4.00571, 0.}, {1.67405, 7.28757, 1.}} | {44.3411, 72.8164, 64.} | inf | 1325. | 385.835 | {224.453, 535.988} | 70.8804 | 0.559818 | newly | 562. | 635. | {486., 804.} | 18.1779 | 0.0705334 | Reff | 2.13223 | 1.3289 | {1.25714, 1.43925} | 37.6757 | ar | 9301. | 5862.32 | {5054., 6875.} | 36.9711 |
| 37 | R0 | {2.78161, 16.0136, 0.1} | {1.73021, 5.85074, 0.02} | {{1.24751, 2.5364, 0.}, {2.22376, 9.35432, 0.}} | {37.7984, 63.464, 116.} | inf | 1098. | 432.618 | {102.183, 762.832} | 60.5995 | 0.532998 | newly | 605. | 636.03 | {316., 807.} | 26.6165 | 0.131972 | Reff | 2.28481 | 1.52376 | {1.13536, 1.91386} | 33.3091 | ar | 9240. | 6183.54 | {3325., 8518.} | 33.0786 |
| 38 | R0 | {2.4454, 8.33501, 0.1} | {2.23728, 8.15724, 0.15} | {{1.71582, 4.02511, 0.}, {2.86001, 13.5542, 1.}} | {15.601, 34.0212, 224.} | inf | 351. | 238.121 | {120.702, 361.033} | 34.4518 | 0.22694 | newly | 491. | 351.99 | {251., 500.} | 31.5988 | 0.179272 | Reff | 2.30772 | 2.13357 | {1.66313, 2.67716} | 14.7158 | ar | 8853. | 8286.71 | {6885., 9236.} | 8.66949 |
| 39 | R0 | {2.9154, 12.3861, 0.1} | {2.55791, 9.63866, 0.18} | {{1.9862, 6.28417, 0.}, {2.80387, 10.6768, 1.}} | {12.262, 22.1817, 244.} | inf | 746. | 622.223 | {427.865, 764.603} | 18.2363 | 0.097758 | newly | 645. | 709.97 | {614., 813.} | 13.5581 | 0.0551022 | Reff | 2.57605 | 2.28842 | {1.84378, 2.47416} | 11.1654 | ar | 9371. | 8907.3 | {8107., 9274.} | 4.94824 |
| 40 | R0 | {2.40923, 15.1963, 0.1} | {1.26181, 3.3359, 0.07} | {{1.22544, 3.07157, 0.}, {1.28596, 3.98513, 0.}} | {47.626, 78.0479, 164.} | inf | 1012. | 217.795 | {137.29, 315.101} | 78.4788 | 0.687387 | newly | 547. | 494.22 | {339., 684.} | 19.6856 | 0.0980767 | Reff | 1.91847 | 1.10483 | {1.07431, 1.12163} | 42.4108 | ar | 8735. | 3883.97 | {3291., 4492.} | 55.5355 |
| 41 | R0 | {2.63137, 15.238, 1.} | {2.38095, 13.2749, 0.04} | {{1.93539, 9.63533, 0.}, {2.6365, 15.4074, 0.}} | {9.61016, 13.4162, 96.} | inf | 1144. | 1093.96 | {650.207, 1336.76} | 15.523 | 0.112491 | newly | 680. | 698.03 | {592., 780.} | 10.5603 | 0.0594263 | Reff | 2.09194 | 1.89538 | {1.62811, 2.04939} | 9.39621 | ar | 9150. | 8525.96 | {7789., 9106.} | 6.84131 |
| 42 | R0 | {2.48769, 19.4593, 0.1} | {2.63793, 19.6307, 0.05} | {{2.66225, 19.9254, 0.}, {2.66225, 19.9254, 0.}} | {7.71401, 3.81367, 146.} | inf | 1677. | 1939.66 | {1915.32, 2026.02} | 18.422 | 0.0784165 | newly | 673. | 768.28 | {738., 805.} | 14.1575 | 0.0572542 | Reff | 1.67844 | 1.70706 | {1.70179, 1.73078} | 2.98177 | ar | 8932. | 9061.9 | {9072., 9186.} | 3.015 |
| 43 | R0 | {2.61199, 10.214, 0.1} | {2.62392, 9.61384, 0.08} | {{1.9862, 6.28417, 0.}, {2.91001, 10.6768, 0.}} | {9.5442, 11.3183, 164.} | inf | 259. | 233.824 | {144.058, 297.019} | 19.0994 | 0.0987535 | newly | 315. | 301.35 | {228., 372.} | 15.5714 | 0.0723081 | Reff | 2.50594 | 2.52248 | {1.93524, 2.78825} | 9.34657 | ar | 9061. | 8968.81 | {7911., 9326.} | 3.96479 |
| 44 | R0 | {2.50994, 18.3382, 0.1} | {1.76188, 9.34252, 0.83} | {{1.58886, 7.29751, 0.}, {2.66225, 19.9254, 1.}} | {31.7458, 51.824, 764.} | inf | 1225. | 678.688 | {442.23, 1627.39} | 55.4522 | 0.351858 | newly | 597. | 567.49 | {486., 666.} | 9.51926 | 0.0432008 | Reff | 1.9314 | 1.43551 | {1.33053, 1.9354} | 25.8039 | ar | 8827. | 6806.93 | {6137., 9125.} | 24.059 |
| 45 | R0 | {2.44283, 16.8338, 0.1} | {2.10217, 12.5334, 1.71} | {{1.25474, 3.26445, 0.}, {2.29887, 14.662, 2.}} | {13.9451, 25.546, 1644.} | inf | 1233. | 933.252 | {225.343, 1155.61} | 24.3145 | 0.182485 | newly | 606. | 606.22 | {558., 662.} | 7.91089 | 0.0376389 | Reff | 1.87707 | 1.66524 | {1.08845, 1.80907} | 11.2854 | ar | 8878. | 7740.06 | {3646., 8680.} | 12.8175 |
| 46 | R0 | {2.51815, 18.1462, 5.} | {2.28302, 15.2072, 0.45} | {{2.11292, 12.9719, 0.}, {2.49092, 17.9391, 1.}} | {9.33742, 16.1965, 91.} | inf | 1241. | 1085.03 | {889.041, 1313.08} | 16.0676 | 0.0794763 | newly | 572. | 591.86 | {550., 628.} | 4.90909 | 0.0206932 | Reff | 1.943 | 1.79374 | {1.69287, 1.91789} | 7.68217 | ar | 8978. | 8535.05 | {8157., 8948.} | 4.96224 |
| 47 | R0 | {2.88885, 16.8987, 0.1} | {1.60521, 5.16088, 0.03} | {{1.44762, 3.83841, 0.}, {1.74592, 5.83416, 0.}} | {44.4342, 70.278, 126.} | inf | 890. | 294.194 | {177.099, 385.26} | 66.9444 | 0.517828 | newly | 505. | 558.37 | {403., 711.} | 21.4911 | 0.0892855 | Reff | 2.47083 | 1.46463 | {1.33262, 1.59301} | 40.7232 | ar | 9337. | 6146.25 | {5322., 7153.} | 34.1732 |
| 48 | R0 | {2.58709, 13.8786, 0.1} | {2.4024, 12.1155, 1.33} | {{1.74092, 5.83416, 0.}, {2.96398, 17.7348, 2.}} | {13.9599, 25.8605, 1262.} | inf | 706. | 619.466 | {360.454, 819.029} | 22.0834 | 0.117939 | newly | 483. | 502.22 | {430., 573.} | 10.3602 | 0.0433442 | Reff | 2.27612 | 2.12747 | {1.5946, 2.57421} | 12.5748 | ar | 9146. | 8563.1 | {7105., 9374.} | 7.55631 |
| 49 | R0 | {2.42959, 10.7132, 0.1} | {2.47886, 11.0285, 0.78} | {{2.12684, 8.23415, 0.}, {2.75598, 12.7047, 2.}} | {13.0554, 21.3947, 750.} | inf | 413. | 440.163 | {282.671, 554.444} | 24.2309 | 0.111116 | newly | 380. | 419.2 | {346., 503.} | 14.5053 | 0.0580691 | Reff | 2.25199 | 2.28717 | {1.97439, 2.51985} | 12.0633 | ar | 8863. | 8713.72 | {8231., 9214.} | 6.02618 |
| 50 | R0 | {2.60513, 13.2796, 0.1} | {2.60857, 15.2445, 0.74} | {{2.14747, 10.7247, 0.}, {2.90165, 18.0404, 1.}} | {7.38053, 21.764, 692.} | inf | 466. | 561.417 | {428.156, 672.083} | 25.3277 | 0.0976691 | newly | 376. | 352.59 | {304., 400.} | 9.0984 | 0.0421336 | Reff | 2.3863 | 2.36227 | {1.98695, 2.60911} | 6.84006 | ar | 9095. | 8997.94 | {8349., 9343.} | 2.84024 |
